# First record of *Panstrongylus megistus* (Burmeister, 1835) (Hemiptera, Reduviidae, Triatominae) from Ilha Grande, Rio de Janeiro, Brazil

**DOI:** 10.1590/0037-8682-0025-2025

**Published:** 2025-09-29

**Authors:** Allan Pitta Seabra, João Paulo Sales Oliveira-Correia, Quezia Moura Oliveira, Maria Clara Rego e Silva, Alena Mayo Iñiguez, Cleber Galvão

**Affiliations:** 1 Laboratório Nacional e Internacional de Referência em Taxonomia de Triatomíneos, Instituto Oswaldo Cruz, Fundação Oswaldo Cruz, Rio de Janeiro, RJ, Brasil.; 2 Laboratório de Parasitologia Integrativa e Paleoparasitologia, Instituto Oswaldo Cruz, Fundação Oswaldo Cruz, Rio de Janeiro, RJ, Brasil.

**Keywords:** Chagas disease, Triatominae, Trypanosoma cruzi, New record, Blood meal source, Kissing bug

## Abstract

**Background:**

Chagas disease is caused by *Trypanosoma cruzi* and transmitted by kissing bugs.

**Methods:**

We investigated the presence of *Panstrongylus megistus* in Ilha Grande, RJ. The insects was analyzed for *T. cruzi* infection and blood meal sources (BMSs).

**Results:**

Morphological identification confirmed *P. megistus*. Molecular analysis revealed *Gallus gallus* as the BMS, with no *T. cruzi* infection.

**Conclusions:**

These findings represent the first record of *P. megistus* in the region, expanding its distribution, highlighting the need for continuous monitoring and implementation of physical barriers to reduce transmission risk. Strengthening health education and surveillance is crucial for preventing the disease.

Chagas disease, also known as American trypanosomiasis, is an infection caused by the protozoan *Trypanosoma cruzi* (Chagas, 1909), that affects approximately seven million people, resulting in approximately 12,000 deaths annually^1^. All triatomines are potential vectors of *T. cruzi* and belong to the subfamily Triatominae, which includes 158 species distributed across 18 genera^2^.

*Panstrongylus megistus* (Burmeister, 1835) was the first triatomine recognized as a vector of the causative agent of Chagas disease^2^ ^,^ ^3^ *.* Although native to the Atlantic Forest biome, this species has been found in other regions, colonizing both domiciliary and peridomiciliary environments, e.g., chicken coops, as well as wild habitats, e.g., opossum and rodent shelters, decaying trees, palm trees, bromeliads, and rock crevices^2^. Before 1930, this species was considered the main domiciliary vector in Brazil, until it was progressively replaced by *Triatoma infestans* (Klug, 1834)^2^. Currently, *P. megistus* has a broad geographic distribution covering Argentina, Brazil, Bolivia, Paraguay, and Uruguay. In Brazil, it is present in all states, except Amazonas, Amapá, Pará, and Roraima^2^.

In the present study, we report the first record of *P. megistus* from Vila Dois Rios, Ilha Grande, municipality of Angra dos Reis, Rio de Janeiro, Brazil (Figure 1).

**FIGURE 1:**
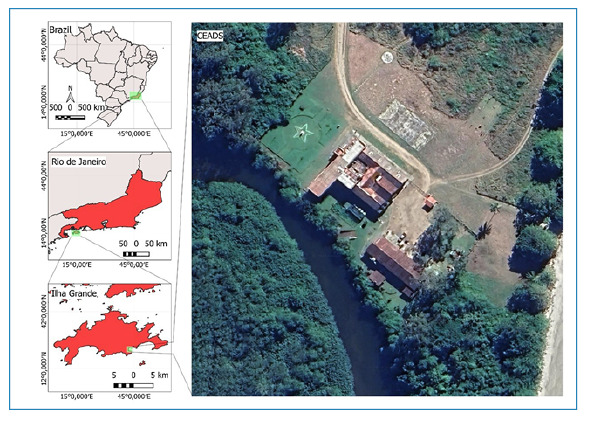
Map showing the location of the “Centro de Estudos Ambientais e Desenvolvimento Sustentável (CEADS)” in Vila Dois Rios, Ilha Grande, Rio de Janeiro, Brazil.

The specimen was found inside the *Centro de Estudos Ambientais e Desenvolvimento Sustentável* (CEADS) (23°24′15′′ S, 43°19′’47′′ W), in the *Universidade Estadual do Rio de Janeiro* (Figure 1). Live specimens were photographed using a smartphone camera (Figure 2). Subsequently, high-resolution photographs were captured using a DFC 295camera (Leica, Wetzlar, HE, GER) coupled to a M205C stereoscopic microscope (Leica), at the microscopy platform of the *Laboratório Nacional e Internacional de Referência em Taxonomia de Triatomíneos, da Fundação Oswaldo Cruz (FIOCRUZ)* (Figure 3). Morphological identification was performed using the dichotomous key of Galvão (2014)^3^, and the specimen was deposited in *Coleção de Triatomíneos do Instituto Oswaldo Cruz (CTIOC)* under code CTIOC Nº 12289.

**FIGURE 2:**
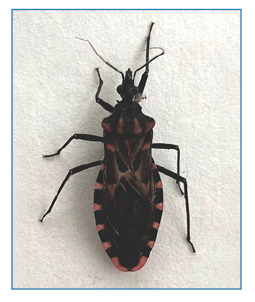
Specimen of *Panstrongylus megistus* photographed a few hours after its collection.

**FIGURE 3:**
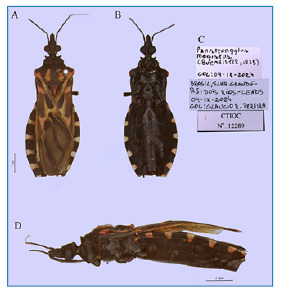
Dorsal **(A)**, ventral **(B)**, label **(C)**, and lateral **(D)** views of a *Panstrongylus megistus* male.

The specimen was dissected to identify the blood meal source (BMS) and trypanosomatid infection, and intestinal contents were extracted. Subsequently, DNA was extracted using physical and chemical digestion, before the application of the DNeasy Blood and Tissue kit (Qiagen, Valencia, CA, USA), following the method described by Pessanha and coauthors^4^. The DNA concentration was estimated by fluorimetry using the QuantiFluor ONE dsDNA kit (Promega, Madison, WI, USA).

BMS was detected using the DNA barcoding approach, targeting the mitochondrial DNA regions 12S rDNA (215 bp) and *cyt*b (358 bp), as previously described^4^. PCR reactions were performed in a final volume of 25 μL, and the reaction mix contained 1X Buffer, 3.0 mM MgCl_2_, 0.2 mM dNTP, 10 μM of each primer, 1U Go*Taq* G2 Hot Start (Promega), and 5.0 μL aDNA (0.5-8.0 ng). To determine whether the specimen was genetically positive for *T. cruzi* infection and to identify the discrete typing unit (DTU) lineages, the assay targeted the 18S rDNA (350 bp) and *cyt*b (200 bp) regions, following the methods described by Borghesan and coauthors and Pessanha and coauthors, respectively^4^ ^,^ ^5^.

PCR products were visualized on 2% agarose gel, stained with GelRed 1× (Biotium, Fremont, CA, USA), and purified with the ExoSAP-IT Express PCR Product Cleanup Kit (Applied Biosystems, Foster City, CA, USA). Sequencing was performed using the Sanger method with a Big Dye Terminator v3.1 Kit (Applied Biosystems) on an RPT01A FIOCRUZ Sequencing Platform (ABI 3730 Sequencer; Applied Biosystems) (https://plataformas.fiocruz.br/unidades/RPT01A). The sequences obtained were initially analyzed using SeqMan v. 7.00 (DNASTAR Lasergene, https://www.dnastar.com/) and BioEdit v. 7.0.4 (Hall, 1999) software, and subsequently compared with the GenBank database using BLAST/ NCBI (Basic Local Alignment Search Tool).

The triatomine captured was a male specimen identified as belonging to the species *P. megistus,* based on the morphological and morphometric characteristics of the individual. *Gallus gallus* (Linnaeus, 1758) was identified as the BMS using the 12S rDNA target, with 98.0% genetic identity (GenBank MT800458). It was not possible to recover a *cyt*b sequence of sufficient quality for a second BMS target. The specimen tested negative for *T. cruzi* infection using the two molecular targets, 18S rDNA and *cyt*b.

This first record of *P. megistus* from Ilha Grande expands our knowledge of the distribution of triatomines on this island. Peixoto and coauthors^6^ have reported the presence of *Panstrongylus geniculatus* (Latreille, 1811) at CEADS and on Praia de Passaterra, Enseada do Sítio Forte, on the portion of the island facing the mainland. Other studies have highlighted the adaptability of *P. megistus* to different habitats^7^.

The genus *Panstrongylus* was established based on a description of the type species *P. guentheri* (Berg, 1879). Since then, several critical taxonomic revisions have been proposed for the genus *Panstrongylus*. For example, *Panstrongylus herreri* and *P. lignarius* were synonymized based on the second internal transcribed spacer rDNA sequences^8^. Garcia and coauthors^9^ examined *Panstrongylus lutzi* (Neiva and Pinto, 1923) specimens from Minas Gerais, Brazil, and identified intraspecific variations in their phallic structures, which aligned with the description of *Panstrongylus sherlocki* (Jurberg et al., 2001). Two recently described species, *P. mitarakaensis* (Bérenger & Blanchet, 2007) and *P. martinezorum* (Ayala, 2009), appear to be closely related to *P. geniculatus*, whereas *P. noireaui* (Gil-Santana et al., 2022) resembles *P. rufotuberculatus*. The most recent taxonomic revision, proposed by Bittinelli and coauthors^10^, based on chromosomal and phylogenetic data, reclassified *Triatoma tibiamaculata* as *Panstrongylus tibiamaculatus* (Pinto, 1926). Currently, this genus comprises 17 species, including one fossil species, of varying epidemiological significance^11^.

The dispersal of triatomines can occur passively, via objects transported by travelers, or actively through adult flight. *Panstrongylus megistus* has limited flight capability, suggesting that its dispersal may be largely passive and possibly facilitated by birds^12^. According to Vilella and coauthors^7^, this species prefers to feed on warm-blooded vertebrates, particularly birds, which is consistent with our findings that *G. gallus* was the BMS of the analyzed specimen. Domestic chickens were observed near the CEADS building.

The negative result for *T. cruzi* infection could be related to the identified BMS, as birds are known to be refractory to trypanosomatid infection. However, DTU TcII *T. cruzi* infection was recently detected by Martínez-Hernández and coauthors (2022) in various organs of the American barn owl, i.e., *Tyto furcata* (Temminck, 1827)^13^. The first acute case of Chagas disease from Rio de Janeiro was identified and confirmed by parasitological, serological, and molecular tests in Mangaratiba, a neighboring municipality on the mainland of Ilha Grande^14^. In addition, *T. cruzi* DNA was detected and characterized (TcI and TcII DTUs) in dogs in this municipality^15^, demonstrating the potential for parasitic infection in the surroundings of Ilha Grande. 

The new record and identification of the BMS of *P. megistus* in this study contributes to the understanding of the geographic distribution and ecology of this insect. As a critical vector of Chagas disease, *P. megistus* requires attention, particularly because it is the second record of an invasive species at the CEADS building. Therefore, we strongly recommend installing physical barriers, such as window screens, to reduce the potential risks to hosts, workers, and students. These measures are highly effective in preventing triatomine bugs from entering living and working spaces. However, fostering collaboration with the community is essential for achieving long-term prevention. This can be accomplished through comprehensive health education programs that raise awareness regarding the disease, its modes of transmission, and effective preventive measures. Additionally, strengthening community surveillance systems is critical for enabling early detection and rapid responses to potential outbreaks. By actively engaging community members in these efforts, we can cultivate a more resilient and well-informed population equipped to sustain effective disease prevention strategies over time.

## Data Availability

Data available upon request.
